# Concluding remarks: culturing cells and engineering tissues for regenerative medicine

**DOI:** 10.3389/fbioe.2026.1882500

**Published:** 2026-07-24

**Authors:** Lucio Luzzatto

**Affiliations:** University of Florence, Firenze, Italy

**Keywords:** bone, burns, cornea, scaffolds, tissue engineering

Regenerative medicine consists of therapeutic procedures that aim to repairing, replacing, or rescuing damaged human cells, tissues, or organs to restore normal function: in common parlance, it is a tall order. In this broad field certain procedures have already become part and parcel of medical practice: autologous skin grafting was developed more than 3,500 years ago (Kohlhauser et al., 2021), and replacing blood through allo-transfusion has been routine for about one century (Harvey et al., 2013). Today we are in an era where allo-transplantation of bone marrow and of several solid organs is widely practiced, with a success rate, at 1 year, of around 90% or more, and variable success rates subsequently.

In regenerative medicine, between the auto-approach and the allo-approach there is a major difference from the immunological point of view. Indeed, a major clinical challenge with every allo-transplantation is the need to prevent rejection, and in many cases also to prevent graft-versus-host disease: both of which require intervention, usually by the use of immuno-suppressive drugs, and sometimes also radiation. In this respect to treat a disease or an injury by harnessing only the patient’s auto-repair mechanisms, e.g., by using the patient’s own stem cells, perhaps in combination with other devices, means to confront a different sort of challenge: I daresay this has been the leitmotif of Ranieri Cancedda’s path over the 60 years of his scientific life, that well deserves being celebrated in this issue of Frontiers in Bioengineering and Biotechnology.

Disease conditions are widely variable in aetiology and in pathophysiology, but they have at least one thing in common: since our body consists of cells and of extracellular structures, it is almost certain that both are involved. The same is true when we consider the ageing process: although the limelight has been often on stem cells and on cell renewal, in advancing age consistent findings are loss of elastic fibres and atheromatous vessels. Therefore regenerative medicine must deal both with cells and with extracellular structures, and Ranieri has underscored the importance of both throughout his career: thus, he has worked on the biological potentials of mesenchymal cells (Tasso et al., 2013), and of fibroblasts (Martin et al., 1997); but also on artificial materials that can be used in therapeutic procedures in humans. There is no doubt that achieving repair of large bone defects through the combined use of autologous bone marrow stromal cells and macro-porous hydroxyapatite scaffolds has been a landmark in his itinerary: the Letter to the Editor reporting this procedure (Quarto et al., 2001) has had more than one-thousand citations! And this was not the only ‘first’ of Cancedda’s group: they were also able to correct hypospadia by autologous grafts of cultured urethral epithelium (Romagnoli et al., 1990), and to restore vision in patients with severe corneal damage by using cultures from autologous progenitor cells localised in the limbus (Pellegrini et al., 1997).

In the area of regenerative medicine, perhaps more than in others, completing the path from experimentation to therapy needs imagination; and obtaining approval by regulatory bodies needs resilience in the face of trial and error iterations. Indeed, even procedures that appear promising have not yet been approved (Hollister and Murphy, 2011): but it is encouraging that in 2021 FDA has approved StrataGraft, that combines living cells and a structural scaffold, and the same has been granted orphan designation in Europe as an Advanced Therapy Medicinal Product. Stratagraft is used for the treatment of deep partial-thickness burns, another highly demanding intervention for which Cancedda’s group has carried out pioneering work (De Luca et al., 1992a). For musculo-skeletal tissue engineering, 3D printed biodegradable scaffolds are regarded as promising, as they are based on polycaprolactone, a polymer that is already FDA-approved (Lui et al., 2026).

Healing of large bone losses and of severe burns can be regarded as paradigmatic of how stem/progenitor cells and scaffolding materials can be integrated for the purpose of regenerative medicine (Bhumiratana et al., 2016): but this general concept can be applied in other areas as well, particularly skin (MacNeil, 2007), cartilage (Makris et al., 2015), and within the cardiovascular system (Maiullari et al., 2018): Cancedda’s group has been active in all of these. They have carried out early investigations of the biology of keratinocytes (Barreca et al., 1992), and they have promoted progress in the management of chronic leg ulcers (De Luca et al., 1992b). With respect to cartilage, Ranieri’s group have shown that in primary cultures of human articular chondrocytes platelet lysates are able to activate chondro-progenitor cells, meaning that stem cell niches become activated: thus the regeneration of cartilage can be improved (Carluccio et al., 2020). As for the cardiovascular system, providing a functional vascular network is a major challenge in tissue engineering: work from Cancedda’s lab has shown that amniotic fluid cells secrete pro-angiogenic factors as well as mediators of neo-arteriogenesis, that may be crucial in tissue repair and regeneration (Mirabella et al., 2011).

It is generally understood that basic research is driven by curiosity, as it aims to discover something that is fundamentally new, whereas applied research aims to solve specific practical problems: in the mind of many there is an undeniable if unstated notion that the former is somewhat superior to the latter. I think that precisely in order to counter this notion the phrase ‘translational research’ has been appropriately coined, and has become a staple of scientific discourse especially in the biomedical area: translational research aims to turn basic scientific discoveries into applications that directly improve human health. Regenerative medicine is a prime example of translational research and it is, in many ways, more difficult than basic research, because it requires a thorough understanding of the cellular and molecular controls of cell proliferation and differentiation, of the physiological responses to tissue injuries, and of the healing process: but at the same time, it requires a thorough command of contemporary tissue engineering techniques and products, and of tissue reconstruction and repair technologies. I cannot help recalling that a Nobel laureate once told me that he thought, against current trends, that the only adjectives needed to qualify research are ‘good’ versus ‘poor’. I think Ranieri Cancedda’s lifetime work is an eminent example of good translational research.

If I am allowed to conclude on a personal note, I feel glad and somewhat proud that, with both Ranieri Cancedda and Fiorella Descalzi Cancedda we have been collaborators, early on in their careers (Cancedda et al., 1973)^;^ (Meloni et al., 1980), and also subsequently (Banfi et al., 2002). The relationship between genetic inheritance and cultural transmission in the human species is complex (Cavalli-Sf et al., 1983): be that as it may, of their children Corrado is a dedicated global health physician (Cancedda et al., 2026), and Laura a dynamic neuro-scientist (Rastogi et al., 2024). I am thankful for having been allowed to contribute to a celebration of the first 60 years of Ranieri’s scientific life.
